# A review of the therapeutic and biological effects of edible and wild mushrooms

**DOI:** 10.1080/21655979.2021.2001183

**Published:** 2021-12-07

**Authors:** G Anusiya, U Gowthama Prabu, N V Yamini, N Sivarajasekar, K Rambabu, G Bharath, Fawzi Banat

**Affiliations:** a Department of Biotechnology, Kumaraguru College of Technology, Coimbatore, India; b Department of Chemical Engineering, Khalifa University, Abu Dhabi, United Arab Emirates

**Keywords:** Mushroom, nutraceuticals, fungal biomass, pharmacological properties, functional foods, active compounds

## Abstract

Throughout history, mushrooms have occupied an inseparable part of the diet in many countries. Mushrooms are considered a rich source of phytonutrients such as polysaccharides, dietary fibers, and other micronutrients, in addition to various essential amino acids, which are building blocks of vital proteins. In general, mushrooms offer a wide range of health benefits with a large spectrum of pharmacological properties, including antidiabetic, antioxidative, antiviral, antibacterial, osteoprotective, nephroprotective, hepatoprotective, etc. Both wild edible and medicinal mushrooms possess strong therapeutic and biological activities, which are evident from their *in vivo* and *in vitro* assays. The multifunctional activities of the mushroom extracts and the targeted potential of each of the compounds in the extracts have a broad range of applications, especially in the healing and repair of various organs and cells in humans. Owing to the presence of the aforementioned properties and rich phytocomposition, mushrooms are being used in the production of nutraceuticals and pharmaceuticals. This review aims to provide a clear insight on the commercially cultivated, wild edible, and medicinal mushrooms with comprehensive information on their phytochemical constituents and properties as part of food and medicine for futuristic exploitation. Future outlook and prospective challenges associated with the cultivation and processing of these medicinal mushrooms as functional foods are also discussed.

## 1. Introduction

Historically, medicinal mushrooms have exhibited a profound health-promoting ability, which is now evaluated by the medical efficacy of their identified bioactive molecules. For example, the presence of essential and supplementary amino acids, together with organic compounds such as ectin, adustin, ribonuclease, and nicotine, have attracted the use of mushrooms and their extract in cancer therapy [1,2]. Edible mushrooms have a highly desirable taste, aroma, texture, and flavor, while uncultivated medicinal mushrooms have been used mainly in health care for the treatment of simple ailments to pandemic diseases [3,4]. The complex anticancer potential of mushrooms is mainly based on the different groups of characteristic compounds that interact with malignant tissues and cells through a wide range of biological processes to produce therapeutic results [3]. The features of fungotherapy for tumor treatment are usually studied by identifying the metabolism of cancer susceptible cells to fungi-based bioactives [5].

For many centuries, mushrooms have played the role of food and medicine, even before thorough investigation and authentication of their functions. In general, mushrooms are macrofungi that act as a dietary component of food with increased nutraceutical and medicinal properties. In recent years, commonly identified mushroom species such as *Agaricus sp*.(button mushroom),*Cordyceps sp*. (caterpillar mushroom),*Ganoderma sp*. (hemlock varnish shelf), *Grifola sp*. (maitake), *Hericium sp*. (lion’s mane mushroom),*Lentinus sp*. (giant panus), and *Pleurotus sp*. (oyster mushroom) are commercially grown. The main phytoconstituent in the fruiting bodies of mushrooms falls primarily under the polysaccharide compound class and also includes substantial amounts of fibers, other saccharides, terpenoids, and proteins [6]. The micronutrients present in mushrooms include mineral elements such as copper, iodine, iron, potassium, selenium, and zinc, along with the vitamin groups and amino acids present in trace amounts [7]. These constituents regulate growth, fluid balance, and bone health in humans, as well as serve as efficient nutraceuticals to supplement immune responses [8,9]. Furthermore, surplus levels of the secondary metabolites in mushrooms are responsible for their various medicinal properties, such as antioxidant, anti-inflammatory, anticancer, antihyperlipidemic, immunoregulatory, and cardioprotective properties [10]. In addition, mushrooms are rich sources of carbohydrates and proteins along with small amounts of fat content. They are rich in ergosterol and unsaturated fatty acids with bioactive compounds like β-glucans, triterpenoids, antioxidants, etc., making mushrooms a highly nutritious food for humans [11]. These fungi serve as a diet source for high potassium and iron with low sodium, compared to any other food source [12]. Also, the control of blood lipids and glucose levels by non-starch polysaccharides, sclerotium, and the dietary fibers show close association in the betterment of the immune system [13].

Medicinal mushroom variants with the ability to treat and cure ailments such as cold, cough, influenza, asthma, cancer, gastric, and hepatic disorders have been widely reported [14]. Ethnopharmacological studies revealed documentation of the traditional use of mushrooms based on which new pharmaceutics can be developed with the baseline information provided in that study [4]. Currently, the identification, isolation, and designation of the primary bioactive polysaccharides present in mushrooms are extensively researched. These compounds are purified and tested as antitumor and immunostimulant agents in humans through clinical trials. Approximately 126 medicinal functions of mushrooms, derived from both wild and edible types, have been successfully studied. This highlights the need for increased cultivation of medicinal mushrooms considering their high therapeutic potential [15]. The abundant antioxidant activity found in both wild and edible mushrooms is mainly due to the presence of bioactive constituents such as phenols, flavonoids, vitamins, tocopherols, and carotenoids that could be further exploited for the prevention of free radical-related diseases [16].

Thus, consolidative information on the various functional and medicinal properties of edible and wild mushrooms would serve as a vital source for researchers and scientists in the field of mushroom cultivation, food processing, and healthcare. In recent years, there has been an increased quantum of research works on mushrooms for the selective extraction of bioactive compounds that could stimulate and enhance the response of human immune cells. Besides, a large number of wild variants of mushrooms known for their various therapeutic values have been discovered in the last five years. A comprehensive summary of the medicinal and biological effects of edible and wild mushrooms is missing, which is highly required for researchers and scientists working in the field of mushrooms. This review provides a clear picture of commercially grown edible mushrooms and the medicinal, nutraceutical, and pharmacological properties of various edible and medicinal wild mushrooms under study. Also, this review would serve as a critical reference for research works focused on the recent trend of medicinal mushrooms cultivation using solid waste-biomass substrate and liquid culture fermentations.

## 2. Edible mushrooms

Edible mushrooms are considered as delicacy food components owing to their attractive culinary and sensory characteristics. In general, the cultivation of edible mushrooms is practiced worldwide due to the striking advantages of low resource requirement and ease of culturing. Recently, edible mushrooms have become increasingly attractive as functional foods due to their salient effects on human health. On the other hand, around 1–3% of the overall human population are allergic to mushrooms which may be due to allergens like spores or oral intake [17]. Most edible mushroom variants are commercially grown or consumed from their wild vegetation. Table 1 tabulates some of the popularly known edible mushrooms grown worldwide for their nutritional and medicinal values.

**Table 1.** List of some edible mushrooms with their medicinal properties

S.No	Botanical Name	Common Name	Geography	Uses	Reference
1	*Agaricus bisporus*	Button Mushroom	Europe and North America	Anti‐tumor, Antioxidant, Antiviral, hypocholesterolemic, Hypoglycemic, Anti-bacterial, Anti-aromatase activity, Anti-proliferative activity, and Proapoptotic property	[158–160]
2	*Agaricus blazei*	Murrill’s Agaricus God’s Mushroom Cogumelo do Sol’ in Brazil, or ‘Himematsutake’ in Japan	Brazil, China and Japan	Atherosclerosis, hepatoprotective, Hyperlipidemia, Diabetes, Dermatitis, Antitumor, immunostimulatory	[161,162]
3	*Agrocybe aegerita or Cyclocybe aegerita*, *Agrocybe cylindracea, or Pholiota aegerita*	Chestnut mushroom/Velvet pioppino Popular mushroom	Europe, United States, Chile, Japan, Korea, China, and Australia.	Nutrition, Alkaloids, Antibacterial, and Immunity	[57,163]
4	*Auricularia auricula-judae*	Jew’s ear, (black) wood ear, jelly ear	China and Australia	Antioxidant, Antitumor, hypolipidemic, Anticoagulant, and Immunomodulatory	[164–166]
5	*Auricularia polytricha*	Cloud Ear Fungus/Jew’s Ear mushroom	India and China	Antitumor, and Anti-hypercholesterolemic	[167,168]
6	*Boletus edulis*	Penny Bun/ King Bolete (Fungi) Cep	Europe, Asia, North America, south Africa, Australia, New Zealand, and Brazil	Anti-neoplastic, and Anti-oxidant	[169,170]
7	*Calbovista sculpta*	Sculptured puffball, Sculptured giant puffball	North America and Brazil	Therapeutic properties	[171]
8	*Calocybe indica /* *Tricholoma giganteum*	Milky mushroom	India	Prebiotic, Antiproliferative and Immunomodulatory	[172,173]
9	*Calvatia gigantea*	Giant puffball	Britain, Ireland, and North America	Antioxidant, Antitumor, Antibiotic, Antiviral, and Antibacterial	[174,175]
10	*Cantharellus cibarius*	Girolle	France, Britain and India	Hepatoprotective, Antimicrobial, Antioxidant, Antihypersensitive, Antiinflammatory, and neuroprotective	[176,177]
11	*Clitocybe nuda*	wood blewit	Britain, Netherland, France and California	Antimicrobial, Anti-diabetic, hypolipidemic, Antihyperlipidemic, and Antitumor	[178,179]
12	*Cordyceps militaris*	Caterpillar fungus	Nepal, China, Japan, Bhutan, Korea, Vietnam, and Thailand	Insulin resistance, Effect on angiogenesis and tumor growth, Hypoglycemic activity, Immunity, and Antioxidants	[180,181]
13	*Cortinarius caperatus*	Gypsy mushroom	Northern regions of Europe and North America.	Antifungal, Antioxidant, and Antiviral	[182,183]
14	*Craterellus cornucopioides*	black chanterelle, black trumpet, trumpet of the dead.	North America, Europe, Japan, and Korea	Antioxidant, Anti-hyperglycemic, Anti-inflammatory, Antimicrobial, and Immunomodulatory	[184,185]
15	*Craterellus tubaeformis*	yellow foot, winter mushroom, or funnel chanterelle	Northern America, Europe, and Asia	Anti-inflammatory and Antioxidant	[186,187]
16	*Flammulina velutipes*	Enokitake velvet shank, golden needle mushroom, or winter mushroom	China, Japan, Korea, and Taiwan	Antioxidant, Anticancer, Antihypertensive, Antimicrobial, immunomodulatory, and hepatoprotective	[188,189]
17	*Ganoderma lucidum*	Lingzhi (Ling chih)/Reishi	Malaysia, China, Japan and South Korea	Antioxidant, antitumor, Anti – androgenic and cardioprotective	[190]
18	*Grifola frondosa*	Hen of the Wood/Maitake	Japan, China Europe and North America	Antioxidant, Anti-diabetic, Anti-tumor, Immunomodulatory, antimicrobial, and hepatoprotective	[116,191]
19	*Gyromitra esculenta*	Brain mushroom, turban fungus, elephant ears, beefsteak mushroom	Britain, Ireland, Europe, and North America	Antioxidant, Anticholinesterase, and Antimicrobial	[192,193]
20	*Hericium erinaceus*	Monkey head or bearded tooth or Lion’s Mane Mushroom	Britain, Europe, central and southern France, North America	Anticarcinogenic, Antibiotic, Antidiabetic, Antifatigue, Antihypertensive, Antihyperlipodemic, Cardio, Hepato, Nephro, and Neuroprotective	[47,194]
21	*Hydnum repandum*	Hedgehog Mushroom or Sweet Tooth or hedgehog mushroom	Ireland, Europe, North America	Antimicrobial, Antitumor, Antibiotic and Cytotoxic	[105,195]
22	*Hypomyces lactifluorum*	Lobster mushroom (Fungi)	North America	Antioxidant	[196,197]
23	*Hypsizygus tessellatus*	Beech Mushroom, Buna shimeji	Europe, North America and Australia	Anti-oxidant, Anti-inflammatory, Anti-allergic, Anti-tumor, Antibacterial, Antifungal, Anti-oxidant and Immunomodulatory	[198,199]
24	*Lentinula edodes*	Shiitake	Asia, China and Japan	Antifungal, Antibacterial, Anti-tumor, cytotoxicity assay, Apoptosis and Antioxidant	[200,201]
25	*Lactarius deliciosus*	saffron milk cap and red pine mushroom	Europe, North America, Central America, Australia, and New Zealand	Antibacterial, Antifungal, Cytotoxic, Anti-inflammatory, Insecticidal, Nematocidal, Antioxidant, Antitumor and Immunomodulatory	[202,203]
26	*Morchella esculenta*	Yellow Morel	North America, North Canada, and India	Antioxidant, Immunomodulatory, Antitumor, Antifungal, and Hepatoprotective	[119,204]
27	*Phellinus linteus*	Mesima	Japan, China, Korea and India	Anti-inflammatory, Anti-tumor, Immunomodulatory, Anti-diabetic, and Antifungal activity	[205,206]
28	*Phellinus rimosus*	Cracked-cap Polypore	Eastern and North America	Anti-oxidant, Anti-inflammatory, neuroprotective, and Antihepatotoxic	[207,208]
29	*Pleurotus eryngii*	King oyster mushroom	Europe, West Asia and North Africa	Therapeutic, Antitumor, and Antimicrobial	[209]
30	*Piptoporus betulinus*	Birch bracket mushroom	Europe, North America and Asia	Anti-viral, Antitumor, and cosmetic use on skin and hair	[210,211]
31	*Pleurotus ostreatus /* *Pleurotus pulmonaris*	Oyster Mushroom	Worldwide	Immunomodulatory, Anti-tumor, hyperglycemia, Anti-oxidant, Ani-viral, Antibacterial, and Antifungal	[212,213]
32	*Russula delica*	Milk-white brittlegill	Europe and Asia	Anti-inflammatory, Antioxidant, Antiproliferative and Antiviral	[214,215]
33	*Schizophyllum commune*	split gill fungus	North east India	Antitumor, Immunomodulatory, Antioxidant, Antifungal, Antineoplastic and Antiviral	[216,217]
34	*Sparassis crispa*	Cauliflower mushroom	Korea	Wound healing in diabetes mellitus, Anti-tumor activity, Immunomodulatory, and Antifungal	[99,218]
35	*Stropharia rugoso annulata*	wine cap stropharia, garden giant, burgundy mushroom, or king stropharia	Europe North America, Australia, and New Zealand.	Antihyperglycemic, Antioxidant, Antibacterial and hypoglycemic	[219]
36	*Tricholoma giganteum*	Matsutake	India	Apoptogenic, Antioxidant, Anticancer and hepatoprotective	[220,221]
37	*Tricholoma matsutake*	Matsutake	Asia, Europe North America Japan, Korea, and China	Immunostimulator, Antioxidant, Antitumor and Anti-microbial	[222,223]
38	*Tremella fuciformis*	Snow mushroom/Silver Ear mushroom/ White jelly fungus	Asia, America, sub-Saharan Africa, southern and eastern Asia, Australia, and Pacific Islands.	Hypoglycemic effect, Hypocholesteromic effect, Antioxidant activity, and Anti-inflammatory	[224,225]
39	*Tuber aestivum*	Burgundy truffle	Europe	Antioxidant, Anti-inflammatory Antiviral, Antimicrobial, Hepatoprotective and Anti-mutagenic	[226,227]
40	*Tuber melanosporum*	Black truffle	Spain, France and Italy	Anti-fatigue, Antitumor, Antioxidant and neurotropic	[77,228]
41	*Volvariella volvacea*	Paddy straw mushroom	Malaysia, China and India	Anti-tumor and Immunomodulatory	[229,230]

**Figure 1.**
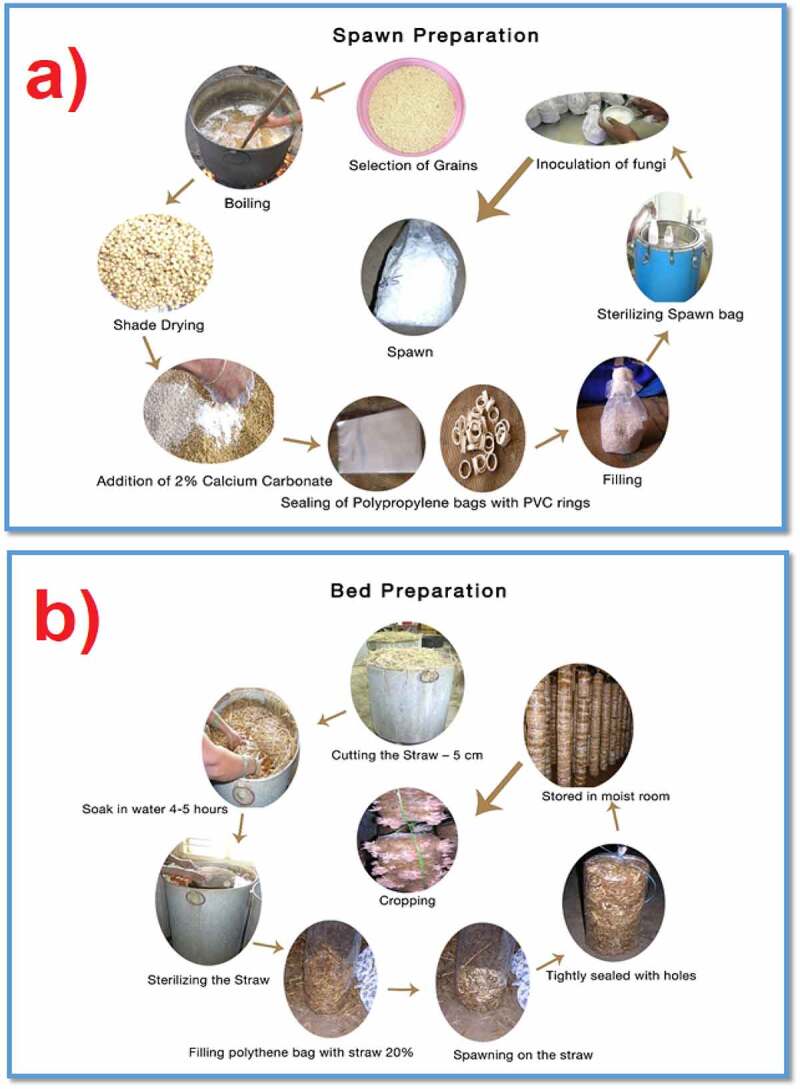
(a) Preparation of spawn and (b) preparation of bed for the cultivation and production of edible mushrooms

Before the commercial cultivation of any edible mushroom, the silhouette of the nutrients and the phytochemical screening of those mushrooms are investigated. For example, the proximate analysis of oyster mushrooms showed the presence of approximately 91% moisture, enriched with proteins, carbohydrates, other macro and micronutrients, and all vitamin groups. Furthermore, phytochemical screening provides critical information on bioactive secondary metabolites such as alkaloids, flavonoids, glycosides, polyphenols, saponins, tannins, and other reducing compounds present in mushrooms [18]. These analyses offer a fair idea of the various resources and cultivation conditions required for the specific cultivation of mushrooms. The commercial process of growing edible mushrooms involves two main stages, spawning preparation, and bed preparation, as shown in Figure 1.

### 2.1 Commercially cultivated mushrooms

*Agrocybe aegerita*, an agaricomycetes fungus, is one of the most popular mushroom types commercially cultivated for its culinary use. The ease of cultivation of this fungus in a solidified medium is an important parameter that makes *A. aegerita* a suitable type for investigating the developmental biology of mushrooms, by which *Pleurotus pulmonarius* and *Pleurotus sajor-caju* were cultivated based on the differences observed in traditional mating experiments [19,20]. Hot water extracts of *Agaricus bisporus* (AbHW) and *Ganoderma resinaceum* (GrHW) mushroom varients, which are cultivated in northern Serbia, possess medicinal values such as peroxidation, antitumor, and antiproliferative activity. The effectiveness in the biological activities of the selected mushrooms provides direction for their use as functional foods [21].

Some upsides of commercially cultivated mushrooms over other variants are their ability to produce the fruiting body in a shorter amount of time, the ability to rapidly produce mycelium in liquid culture, and the ability to manipulate the culture medium to produce optimal quantities of antioxidant and antitumoral compounds. However, the nutritional content and bioactive characteristics of these mushrooms types are found to be affected as a result of postharvest preservation techniques [22].

### 2.2 Commercially harvested wild mushrooms

Wild mushrooms are the variants of fungi that grow invariantly in wild environments and extreme climatic conditions. Owing to their safe and nutritive characteristics, wild edible species are cultivated nowadays on a large scale once their nutritional properties are identified and reported. For example, the wild edible mushroom *Cantharellus cibarius*, which occurs predominantly in Antalya (Turkey), is known for its potent total antioxidant status (TAS), total oxidant status (TOS), and oxidative stress index (OSI) that are evaluated by scavenging activity of 2,2-diphenyl-1-picryl-hydrazyl-hydrate (DPPH) [23]. *Termitomyces heimii*, an edible mushroom grown in sub-Saharan Africa, is one of the most consumed wild mushrooms known for its taste, flavor, and medicinal properties equivalent to those of meat and fish. The flour of this fresh mushroom was identified with proteins, fats, moisture, ash, carbohydrate, and high energy values. *T. heimii* was suggested for consumption due to its high protein and low-fat content (3.58%) and also its good foaming characteristics with essential functional properties, making it a suitable ingredient in food industries [24,25].

Owing to the recent increase in the demand among consumers for these wild edible mushrooms, these wild mushroom variants are commercially cultivated under their native conditions through the involvement of local communities. Thus, this approach provides an assurance of livelihood to the local societies and meets the public demand for wild edible mushrooms. In addition, in order to improve the commercial scope of wild edible mushrooms, detailed studies on the toxicity and antinutritional factors present in wild mushrooms must be carried out to establish a complete database on the nutritional supremacy of these wild edible mushrooms [26]. Environmental suitability, raw material quality, variable process cycle, need for constant monitoring, and insects/pests’ attacks are the prime challenges faced while cultivating the commercial wild species of mushroom. Besides, health complications caused by mushroom spores to cultivators, competition with other food products, and technology/training deficit challenge the large-scale cultivation of wild mushrooms [27].

## 3. Nutritional and bioactive compounds in mushrooms

Mushrooms are the higher-class flora of the fungi family and are widely used for human consumption. Approximately 14,000 species of mushrooms have been discovered so far, of which around 2,200 species are identified as edible mushrooms. Among these, about 650 species have been widely studied, cultivated, and consumed for health and medical applications. For example, India alone has 850 native species of mushrooms, which have been investigated for their nutritional and ethnomedicinal uses and their prospective applications in food and pharmaceutical portfolios [28]. Of the 1,500 species of mushrooms found in Japan, the gene pooling technique was used to extract the full potential of wild and edible mushrooms for the development of nutritional and pharmaceutical products. In countries like Nigeria, *Lycoperdon pusillum* and *Calvatia gigantea* (Puff balls) mushrooms are used to treat abrasions, sores, deep cuts, hemorrhage, and urinary infections.

*Boletus edulis, Lentinus edodes*, and *Xerocomus badius*, the three edible mushroom species found in Poland, were analyzed for their phenolic compounds and tocopherols and their relative antioxidant activity. The results showed that carbohydrates were present in abundance in these mushroom types, followed by proteins, ash, and fatty acids (polyunsaturated fatty acids were higher than saturated fatty acids). Besides, the α-, β- and γ-tocopherols were also identified and quantified in mushrooms [29]. Three new strains of oyster mushrooms, *Pleurotus highking, Pleurotus ostreatus*, and *Pleurotus geesteranus*, were developed in Bangladesh and compared for their growth, yield parameters, and chemical composition. Each strain exhibited its own characteristic features and recorded rapid growth, surplus flesh in fruiting bodies, rich chemical composition with high biological activity, and economic culture. The desirable results favored the commercial production of these strains in Bangladesh [30]. The chemical composition of commercially cultivated mushrooms in Italy, namely the *Boletus* group, *Agrocybe aegerita*, and *Pleurotus eryngii, was* examined. Nutritional compounds such as dietary fiber, chitin, beta-glucans, and total phenols were present in both raw and cooked samples. Total proteins were higher in the *Boletus* group than in *Agrocybe aegerita* and *Pleurotus eryngii*, while the quantity of beta-glucans varied between *Boletus* species [31].

Identification, cultivation, and commercialization of new types of mushrooms that can act as a nutraceutical, food, and pharmaceutical ingredient, considering global factors such as food nutrition, health care, environmental conservation, and socioeconomic changes to supplement existing food and medicinal products is a promising research area for engineers and scientists [32]. Notably, mushroom mycelia, which consists of numerous bioactive compounds, appears to be a propitious source for the pharmaceutical industry to produce novel drugs for a wide range of applications. Chitin, dietary fiber, and its hydrolyzed form, glucosamine, which act as valuable food supplements against osteoarthritis, are found in large amounts in mushrooms [33,34]. In general, the polysaccharides in mushrooms carry vital biological information from fungi due to their strong potential for structural variability [35]. In particular, polysaccharides such as hemicellulose, β and α-glucans, mannans, xylans, and galactans serve as prebiotics that stimulates the growth of the gut microbiota of the host by inhibiting exogenous pathogens, thus improving host health [5]. Usually, the dry matter of mushrooms contains low bioactive compounds compared to fresh matter. The main carbohydrates present in the dry matter include chitin, glycogen, polysaccharides, trehalose, and mannitol with β-glucans. Microelements such as potassium, cadmium, and mercury could also be seen in selected species of mushrooms. Other chemicals like ergosterol, provitamin D₂, and phenolics constitute nutrient bioavailability in mushrooms [36]. Important properties and applications of bioactive fungal polysaccharides derived from the *Basidiomycetes* family have been reported through a comprehensive study on the application of these biopolymers as nutraceuticals for human health [37].

Medicinal mushrooms with added nutraceutical values are considered an unparalleled source of healthy foods and drugs. The extracts of the medicinal mushrooms are used to treat patients by incorporating them as food supplements [15]. Bioactive compounds derived from these mushrooms are being used in clinical trials for the treatment of various diseases and infections. It is necessary to screen for toxins present in wild poisonous mushrooms and to adopt the proper methods for the cultivation, processing, preservation, storage, and consumption of edible mushrooms [38]. In general, mushroom proteins have a large amount of biologically active components, commonly classified as laccases, lectins, fungal immunomodulatory proteins (FIP), ribosome-inactivating proteins (RIP), ribonucleases, and other proteins [39]. Comparison of chemical composition between different types of mushrooms such as *Agaricus bisporus, Flammulina velutipes, Lentinula edodes, Pleurotus eryngii*, and *Pleurotus ostreatus* revealed that all of these types possessed higher levels of macronutrients such as proteins, sugars, fatty acids, and tocopherols along with moisture, ash, carbohydrates, and energy values [40].

The protein content of *Pleurotus* mushrooms (20–35%) was found to be higher than the content of cereals (wheat and maize, 10–15%) and comparable to the protein of legumes (peas and lentils, 20–25%). Mushroom proteins showed a deficiency in methionine and cystine, and therefore they can be supplemented with dairy products for a healthy diet [41]. The chemical composition of *Pleurotus ostreatus* samples analyzed by gas chromatography showed the presence of important metabolites such as alcohols, alkanes, amides, esters, fatty acids, terpenoids, and phenols in fresh and dried studies [42]. In a recent study, rice straw used as a substrate for mushroom cultivation was fed to ten beef cattle for two months. A significant increase in body weight and lower levels of blood plasma, cholesterol, and triglycerides were observed in the cattles, indicating the nutritional value of spent substrate feed without abnormal or adverse effects on animals [27]. Multiple beneficial properties of the maize straw substrate used in *Pleurotus ostreatus cultivation* were witnessed when the substrates were utilized as feed supplements for livestock that increased live weight in sheep [43]. A comprehensive study on various bioactive ingredients such as polysaccharides, terpenoids, steroids, phenolics, and alkaloids isolated from mushrooms has been reported for their phytochemical and pharmacological properties [44].

Many species of mushrooms are naturally known for their medicinal importance, such as anticancer, antidiabetic, hepatoprotective, cardiovascular, antitumor, and also as immunomodulatory drugs. *Agaricus* species are used in the treatment of goiter and hormonal imbalances. The development of new pharmacologically active compounds from mushrooms requires the collection and screening of mushroom species cultivated from different regions of the world [45]. *Ganoderma lucidum*, an oriental fungus that contains a greater number of bioactive components, is well known for its therapeutic value and exhibits biological responses that activate the human immune system for numerous defensive functions. The immune-modulating effects of this mushroom are associated with antitumor activities. In addition to this, the species has multiple functions in chemoprevention activity and antioxidant activity, which makes it preferential for tumor inhibitory effects [46].

*Hericium erinaceus*, a medicinal mushroom that is widely consumed in Asian countries due to its rich composition of polysaccharides and a wide range of secondary metabolites, was evaluated in detail for its health-promoting properties [47]. Methanolic extracts of six wild mushrooms, namely – *Lycoperdon perlatum, Cantharellus cibarius, Clavaria vermicularis, Ramaria formosa, Marasmius oreades*, and *Pleurotus pulmonarius* were studied for their phytochemicals composition. The results showed that total phenolics were the main compounds observed in these types of mushrooms, followed by flavonoids and ascorbic acid. The presence of total phenolics directly contributed to the antioxidant and antimicrobial activity of these fungi, which were estimated through studies of minimum inhibitory concentration (MIC) [48].

Among the 2000 edible variants of mushrooms, species such as *Ganoderma* (Reishi), *Lentinus* (Shiitake), *Grifola* (Maitake), *Agaricus* (Himematsutake), *Cordyceps* (Caterpillar fungus), *Pleurotus* (Oyster), and *Hericium* (Lion’s mane) have been thoroughly authenticated for their medicinal uses. Certain bioactive compounds present in these species exhibit anticancer, anti-HIV, and antiviral activities. These components also act as hepatoprotective agents and help to stimulate the human immune system. Nutraceuticals derived from these species help relieve stress and stimulate the immune response of the body when supplemented with a regular diet [8]. The fruiting bodies of the *Pleurotus* genus were studied for their high nutritional and health-promoting value due to their biological macromolecules such as lectins, laccases, RIPs, nucleases, and FIPs which can be attributed as functional probiotic molecules with definitive medicinal values [49,50]. *The fruits of Armillaria mellea* are rich sources of organic acids and alcohols, mainly mannitol, then followed by carbohydrates, ash, fat, proteins, tocopherols, polyunsaturated fatty acids, and other fatty acid groups. Due to the presence of these bioactive compounds, this mushroom variant was concluded as a potent source for nutraceuticals with high medicinal properties [51].

## 4. Pharmacological and pharmaceutical properties

### 4.1 Antioxidant and antitumor activities

Normal cellular metabolism in aerobic organisms produces free radicals such as reactive oxygen species (ROS) and reactive nitrogen species (RNS). An equilibrium between the antioxidant defense system and free radicals must be maintained within the condition of the body to avoid the oxidative stress condition. Natural antioxidant-rich products, which are taken as supplements, help the endogenous defense system reduce oxidative damage. In general, mushrooms contain oxidative resistant constituents such as phenols, flavonoids, tocopherols, carotenoids, and ascorbic acid, that act as a rich source of antioxidants. Flavonoids, a benzo-γ-pyrone derivative, to which hydroxyl groups are added, methylated, sulfated, and glucuronidated during the metabolism to impart catalytic properties to scavenge the ROS and RNS. In the presence of metal ions, ascorbic acid is oxidized to dehydroascorbic acid in the extracellular environment and transported into the cell through glucose transporters for the suppression of ROS and RNS activities. The antioxidant mechanisms of the mushroom species extract can be classified by its bioactive abilities to donate hydrogen, chelate metal abilities, and scavenge superoxide and free radicals [52]. Phenolic compounds are the main category of phytocomponents that are responsible for the antioxidant activity of mushroom species, and their correlation was observed in *Boletus*, a wild edible mushroom. The presence of high levels of phenolic constituents and its extreme antioxidant properties led to the use of *Boletus* as a food source of natural antioxidants [53]. A total of eight medicinal mushroom species from China were evaluated for their antioxidant properties, of which the *G. lucidum mushroom* exhibited the highest antioxidant activity [54]. Evaluation of antioxidant and free radical scavenging activities for Taiwanese mushroom extracts showed that the *Agaricus* variant was superior in antioxidant activity, followed by other types such as *Flammulina, Hypsizigus, Hericium, Lentinula, Pleurotus*, and *Volvariella* [55]. Phenolic antioxidants inhibit lipid oxidation by free radical scavenging activity, where the activity increases with the number of hydroxyl groups associated with the molecules.

In recent times, mushrooms have also been used as antitumor agents and immunomodulators. Single or both enantiomer forms of numerous chiral biomolecules present in mushrooms are responsible for this biological activity. These chiral biomolecules mainly include the proteins, sugars, amino acids, and nucleic acids present in mushrooms. Among various types of mushrooms, *Shiitake* mushrooms contain high levels of sugars and polyunsaturated fatty acids (PUFA) with very little saturated fatty acid (SFA) content. Oyster and King Oyster mushrooms have high concentrations of monounsaturated fatty acids (MUFA) along with PUFA and SFA content in their samples [40]. *Taiwanofungus salmoneus*, a higher basidiomycete used for inoculating cooked grains (wheat, oats, embryo rice), was evaluated for its reducing power, chelating, and scavenging ability. The results showed that the extracts of the fermented products efficiently suppressed the production of tumor necrosis factor-α, interleukin-1β, and interleukin-6 in RAW 264.7 murine macrophage cells [56]. Gargano et al. discussed the phase I clinical trials conducted on humans suffering from different types of cancer by making them consume *A. blazei, G. lucidum*, and *G. frondose* mushrooms, which are known to have antitumor and anticancer properties, thereby proving the safety of mushroom intake [57].

The broth extract of the endophytic fungus *Corynespora cassiicola* L36 consisted of three compounds, namely, corynesidone A (1), B (3), and corynether A (5). The results of the DPPH and oxygen radical absorbance capacity (ORAC) assays showed that the radical scavenging activities of these bioactives were comparable to those of pharmaceutical-grade ascorbic acid [58]. In another study, mushroom extracts isolated using cyclohexane, dichloromethane, methanol, and water were tested for their antioxidant properties. Methanolic extracts of *P. schweinitzii* exhibited the highest antioxidant activity in the DPPH, ABTS (2,2ʹ-zino-bis(3-ethylbenzothiazoline-6-sulfonic acid)) and ORAC assays [59]. The antioxidant activities of *Agaricus bisporus* ethanol extract were evaluated by in *vivo* and *in vitro* methods. For the *in vivo* study, the extract showed its effective antioxidant activity in the serum, liver, and heart of mice. For the *in vitro* antioxidant assay, the extract exhibited greater antioxidant activities against superoxide radicals, hydroxyl radicals, and reduction of ferric ions [60].

*Inonotus obliquus*, a well-known medicinal mushroom for its traditional tumor therapy, was examined for its antioxidant activity. The methanolic extract of the fungus was identified to contain three varieties of inonoblins and three varieties of phelligridins, with a strong scavenging effect against DPPH and ABTS radicals [61]. In another study, methanolic extracts of two wild mushrooms, *Lactarius deliciosus* (L.) and *Tricholoma portentosum* (Fr.) were reported for their high total phenolic content and superior scavenging effects against free radicals through various chemical assays [62]. In addition, the methanolic extract of *Pleurotus florida* was examined for its higher antioxidant activity as established by DPPH, hydroxyl, and superoxide radical assays together with nitric oxide, reducing power, and metal chelating assays [63]. *Pleurotus ostreatus* were reported for their antioxidant abilities, which were identified through the scavenging effects of free radicals. The extract exhibited scavenging capacities of about 55 to 60% in hydroxyl and superoxide radicals for studies carried out at a concentration of 10 mg/mL [64]. Among the different solvent extracts of *Pleurotus squarrosulus*, hot water extraction produced stronger phenolic, flavonoid, β-carotene, and lycopene content with better antioxidant activities [65]. Enzyme inhibition and metal chelation reactions do not affect the reaction when stable DPPH radicals are used [65]. A comparative study on the antioxidant potential expressed in terms of FRAP and % inhibition using a DPPH scavenging assay was carried out along with the quantification of total phenols and half-maximal effective concentration of EC50 in selected medicinal mushrooms. The DPPH radical scavenging activity showed maximum inhibition of about 90 to 98% for *Suillus luteus, Boletus edulis*, and *Amanita rubescens*. The FRAP assay revealed the maximum reducing potential in *Boletus erythropus* (~62 mmol/g), followed by *Suillus luteus* (~58 mmol/g) and *Boletus edulis* (~53 mmol/g). Total phenol quantification followed the order *Boletus edulis* (~13 mg/g) > *Boletus pseudosulphureus* (~11 mg/g) > *Boletus erythropus* (~10 mg/g). The estimation of half-maximal effective concentration expressed as EC50 was dominated by *Pleurotous dryinus* (~25 mg/mL) succeeded by *Lactarius piperatus* (~24 mg/mL) and *Lactarius volemus* (~21 mg/mL) [66]. The aforementioned mushrooms were found to display potent antioxidant activities, making them suitable for a wide range of food, pharmaceutical, and health care applications. Antioxidant replenishment in the body may aid in the prevention of oxidative stress and cancer. According to a new study, 18 g of mushrooms per day may enhance the antioxidant activities of the cell and reduce the risk of cancer [67]. Compared with other similar foods (such as shrimps, tofu, avocados, sunflower seeds, and almonds), mushrooms are highly rich in antioxidant and antitumor components. The minimum essential concentration for the antioxidant and antitumor activities of mushrooms is nearly one-tenth that of the other similar foods as specified [68].

### 4.2 Anti-inflammatory and anti-aging activities

Inflammation is closely associated with the emergence of a number of incurable disorders such as arteriosclerosis, diabetes, neurodegenerative diseases, and cancer. As mushrooms have excellent medicinal and nutritional values, they have been widely used for the treatment of inflammation over thousands of years. Such mushrooms are rich in anti-inflammatory compounds, namely, polysaccharides, phenol, and indole compounds, steroids, fatty acids, carotenoids, vitamins, and metals. Edible mushrooms are used as functional foods due to their antagonistic role against the tumor, virus, cholesterol, blood glucose, and free radicals [69,70].

Anti-inflammatory constituents from *Inonotus obliquus* were isolated on the basis of an effective bioassay method. Extracts obtained using petroleum ether, and ethyl acetate inhibited nitric oxide (NO) and nuclear factor-κB (NF-κB) luciferase activity in macrophage RAW 264.7 cells. From these extracts, six main constituents were isolated and identified for their anti-inflammatory activity on human prostatic carcinoma cell PC3 and breast carcinoma MDA-MB-231 cells [71]. Cordymin, a peptide that has anti-inflammatory properties, was purified from the entomopathogenic fungi *Cordyceps sinensis* and was analyzed for its cytokine levels and antioxidant properties [72]. In another study, β-D-glucan was purified from aqueous (cold and hot water), and alkaline (KOH) extracts containing polysaccharides that belong to the medicinal mushroom *Cordyceps militaris*. Gas Chromatography-Mass Spectrometry (GC-MS) and Nuclear Magnetic Resonance (NMR) spectroscopy confirmed the linear chain structure of the polymer, which was composed of β-D-Glcp (1ʹ3)-linked. Aqueous extracts were found to stimulate the expression of interleukin-1β (IL-1β), tumor necrosis factor-α (TNF-α), and cyclooxygenase-2 (COX-2) by THP-1 macrophages, and the β-(1ʹ3)-D-glucan possessed the highest anti-inflammatory activity among the various compounds observed in *C. militaris* polysaccharide extracts [73]. In addition, the ethanolic extracts (95%) of the fruiting bodies of *Elaphomyces granulatus*, an edible mushroom, were examined for their anti-inflammatory effects. The extract consisted of two active low molecular weight aromatic compounds, namely syringaldehyde and syringic acid, which showed 68% inhibitory activity against the enzyme COX-2 in mice at 50 μg/mL concentration. The purified enzyme showed its activity at 0.4 μg/mL concentration with an IC_50_ of 3.5 [74]. Similarly, the soluble fractions of *Hericium erinaceus* (EAHE) in ethyl acetate suppressed the (TNF))-α and (IL-6) in RAW264 cells. EAHE inhibited c-Jun N-terminal kinase (JNK) activation, which was responsible for inhibiting pro-inflammatory cytokines. Furthermore, the ethanolic extract of *Hericium erinaceus* reduced the accumulation of myeloperoxidase induced by dextran sulfate sodium in colon tissues and weakened histological changes in neutrophils and lymphocytes, and protected the mucosal epithelium, thus acting as a protective agent in the treatment of inflammatory bowel diseases [75,76].

Around 19 fruiting bodies of *Phellinus igniarius* species were isolated and examined using NMR for their structure and pharmacological effects. Two bioactive compounds present in them have been reported for moderate inhibitory effects on NF-κB with their respective fold values of 0.5 and 0.65 in HeLa cells at 100 μmol/L concentration [77]. Agaricoglycerides, a class of fungal secondary metabolites, were administered in mice to stop liver inflammation and correct liver glycemic metabolism dysfunction. The effects of supplements on IL-1β, vascular endothelial growth factor-α, hepatic glycogen, interleukin-17, insulin secretion, TNF -α, adiponectin, leptin, NF-κB activation, and total antioxidant activity were examined. It was observed and concluded that the supplement decreased the level of inflammatory cytokines and inhibited metabolic dysfunction by suppressing the NF-κB pathway [78]. *Agaricus blazei Murill*, an edible mushroom, was examined for its effects against inflammatory processes induced by different agents. Alkaline and aqueous extracts of this mushroom were observed to marginally inhibit edema. Both extracts inhibited the swelling process when the complete Freund adjuvant was used and also helped heal ulcer wounds, and inhibited neutrophil migration. *A.blazei* extracts were recommended as a therapeutic medicine in inflammatory diseases as they activated the immune system and its cells [79]. *Ganoderma lucidum* mycelium ethanol extracts were evidenced to have anti-inflammatory activity against carrageenan-induced and formalin-induced chronic inflammatory paw edema on mouse skin with a performance of 56% and 60% inhibition, respectively [80].

Mushrooms are rich in ergothioneine and glutathione, which promote health and anti-aging potential. About 13 species of the Italian Porcini mushroom were examined and reported for their high levels of ergothioneine and glutathione [81]. Mycelia selenium polysaccharides (MSPSs) obtained from *Agrocybe cylindracea* were purified and characterized for their anti-aging activities *in vivo*. The results showed that MSPS significantly decreased malonaldehyde content and total cholesterol levels. Additionally, a significant improvement was observed in the activity of the superoxide dismutase and glutathione peroxidase enzymes with an improved antioxidant capacity in response to D-galactose-induced aging. Furthermore, the qualitative analysis of MSPS denoted its selenium content and monosaccharide composition consisting of glucose, galactose, mannose, arabinose, and rhamnose. These results showed that these polysaccharides might be suitable for preventing toxic chemical-induced aging processes and can be recommended for anti-aging therapy [82]. Similar to the aforementioned study, a Lachnum extracellular polysaccharide, YM261 (LEPS-1), a glucose linked by the β-(1 → 3)-d-pyran glycosidic bond, was successfully demonstrated for its stimulating action of antioxidant enzymes, such as SOD, catalase, and GSH-Px against the aging phenomenon in d-galactose model mice [83].

### 4.3 Anticancer and anti-aromatase activities

Mushrooms exhibit their anticancer characteristics through their stimulus action on cancer-fighting immune cells called lymphocytes. Different mushrooms belonging to different genus were studied for their antihistamine effects along with their anticancer activity in various forms, such as angiogenesis inhibitor, antimitotic, mitotic kinase inhibitor, oxygen species inducer, and topoisomerase inhibitor, ultimately preventing cancer cell proliferation. A review of the pharmacologically active compounds present in mushrooms that are used in cancer treatment has been reported with insights into the biological mechanisms of their anticancer activity [84].

**Figure 2.**
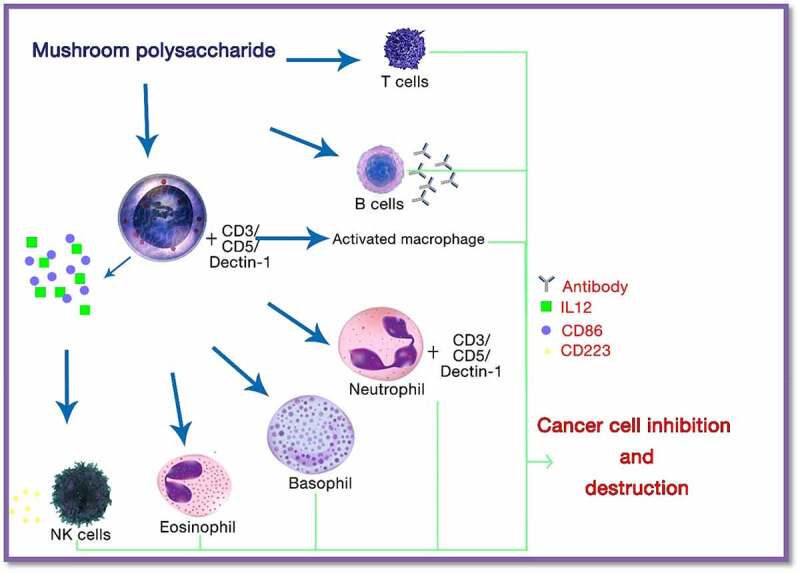
The action of mushroom polysaccharides on the cells responsible for the immune system

Polysaccharides and their derivatives are the major compounds present in mushrooms, which are mainly responsible for anticancer properties. The anticancer potential of these biological macromolecules depends on the origin, solubility, structure, and isolation method. Anticancer properties can be expressed both directly and indirectly by the immune stimulation method and inhibition of cell proliferation or induction of apoptosis, respectively, as shown in Figure 2. For example, in the case of Basidiomycota mushrooms, bioactive compounds such as lentinan, polysaccharide K (PSK), and schizophyllan are the main derivatives that are responsible for the anticancer potential of this mushroom variant [85].

Polysaccharide Krestin (PSK), which was isolated from *Trametes versicolor*, consisted of β-glucan, sugar part, along with a peptide connected by glycosidic bonds. Like lentinan, it is an extremely prevalent drug in Japan for various ailments. Various clinical examinations have demonstrated its competence as a chemotherapeutic drug equivalent and supplement. Alone, as an anticancer medication, it is used in veterinary drugs against sarcoma, carcinoma, mammary malignant growth, colon disease, and malignant lung growth [83]. Two extracts of *Phellinus linteus*, namely PL-ES and PL-I-ES, were tested against 10 different human cell lines. PL-ES (100 µg/mL) exhibited anti-cancer activity, by reducing growth in all cancer cells at the end of 72 h, while PL-I-ES with the same concentration (100 µg/mL) was found to be effective in four cancer cells and an increase in its concentration (250 µg/mL) showed a significant reduction in the other seven cancer cells. Lipid peroxidation (LPO) activity showed the cytotoxic effect and growth reduction of cancer cells by both extracts. In addition, the enzyme assays for Capsace, Csp-3, and Csp-9, and the pro-apoptotic regulators exhibited enzyme activation by bioactives in extracts that led to cell lysis due to oxidative stress in correlation with apoptosis [86].

Chinese medicine emphasizes the use of hot water extracts from fruiting bodies of the genus *Phellinus* for human body refreshment and an increase in life expectancy. In addition, the *Pleurotus* genus is cultivated throughout the world for its antioxidant, antitumor, anti-inflammatory, antihypertensive, and antihypercholesterolemic properties [87]. Methanol extracts of various Oyster mushrooms (based on the color of the strain) were collected and analyzed for their antioxidant and anticancer activities. *The extract of Pleurotus ostreatus* was observed in gray, while the extracts of *Pleurotus cornucopiae* and *Pleurotus salmoneo stramineus* were observed in yellow and pink, respectively. Among these, the yellow strain showed high chelating ability, total phenolic content, reducing power, and radical scavenging activity, which was three times higher than the gray strain. The strains were treated against the human colon cancer cell line HT-29, in which the gray and pink strain extracts exhibited higher inhibitory activities of ~40% than the yellow strain [88].

Mushroom polysaccharides are supplemented in food and medicinal drugs, as they therapeutically help prevent and protect the antagonistic effects of some of the major diseases. These polysaccharides act as prebiotics in the digestive system since the mechanism of action involves the gut microbiota. The *Hericium erinaceus* mushroom is reported to contain more than 50 organic compounds, among which many of them are secondary metabolites related to cancer [89]. *Cordyceps militaris* has been widely used in eastern Asia as a nutraceutical agent and in China to treat cancer patients. This mushroom was studied using anion exchange chromatography with the extract purified from the dried fruiting bodies, called the cytotoxic antifungal protease. The Molecular mass of the protein was analyzed by electrophoresis and was found to be approximately 12 kDa (pI of 5.1). The protease activity was optimal at a temperature of 37°C and a pH of 7–9. Phenylmethylsulfonyl fluoride, a serine protease inhibitor, was used to inhibit enzyme activity. Furthermore, the protein exhibited an antifungal effect against *Fusarium oxysporum* with strong cytotoxicity against human breast and bladder cancer cells [90].

The ethanol extract of *Ramaria flava*, an edible mushroom, was studied for its chemical compositions, antibiotic assay, anticancer activity, and inhibitory effects against tumor cell MDA-MB-231. An IC_50_ value of ~66 µg/mL was observed in all three tumor cell lines tested, and total phenolics in the ethanolic extract varied between four different fractions. The high antibiotic activities of the extract were established against three different strains of microorganisms and fungi through the well diffusion method and the poisoned medium technique [91]. *Agaricus blazei Murrill* extracts were reported for their antimutagenic and anticarcinogenic properties. *The in vivo* assay of the aqueous extracts of *A. blazei* exhibited its antimutagenic potential against DNA damage caused by an indirectly acting alkylating agent, cyclophosphamide [92]. In another study, the fungus *Agaricus blazei* was tested for its anticarcinogenic and antigenotoxic characteristics by inhibiting DNA changes that corresponded to cancer development. The mushroom was supplemented along with a normal diet to test genotoxicity in mice, which ensured the absence of genotoxic effects. It was inferred that the administration of *A. blazei* in any form of extracts annulled any mutagenic activity. The bioactives of *A. blazei* possessed immunomodulatory properties, which activated macrophages and neutrophils, resulting in cancer regression. In addition, *A. blazei* based polysaccharides aided in the stimulation of lymphocytes, ensuring anticancer activity [93].

In general, immune cells or stimulated T cells mediate the antitumor activity of mushroom polysaccharides. Cellular responses such as cytokine expression are triggered by mushroom-derived polysaccharides. The conjugation of polysaccharides with polypeptides and proteins exhibits greater antitumor and anti-aromatase activities [94]. *Agaricus bisporus* (white button mushrooms) has been reported to be a potent chemopreventive agent for breast cancer. The bioactive compounds (such as linolenic acid and conjugated linoleic acid) of this mushroom, derived as ethyl acetate fractions, were capable of inhibiting aromatase with high potency. The conjugated linolenic acid fraction of the mushroom extract was found to inhibit the testosterone-dependent expansion of MCF-7aro cells [95]. *Corynespora cassiicola* L36, an endophytic fungus, contained three types of phytochemical compounds that exhibited powerful radical scavenging activity and ORAC activity, contributing to its antioxidant potential. One of the compounds, corynesidone A (1), was found to inhibit aromatase activity with an IC_50_ value of 5.30 μM [58]. Oral solution of *Poria cocos* polysaccharide was developed to treat various ailments such as hepatitis, cancer, and also for the post-medication effects of chemo or radiation therapy [96]. Thus, it can be concluded that the crude or purified compounds in the mushrooms exhibit potent anticancer and antiaromatase activities when consumed directly or as nutraceuticals along with commercial drugs.

### 4.4 In vitro antimicrobial and in vivo antibiotic activities

Mushrooms could be effectively substituted as a potent antimicrobial against pathogenic microorganisms [97]. *Lentinus edodes* was one of the most explored species with an extensive antibacterial activity against both Gram-positive and Gram-negative bacteria. High- and low-molecular-weight compounds ranging from proteins and peptides to steroids and organic acids present in the mushroom extract are responsible for the antifungal property. Grifolin derived from *Albatrellus dispansus* was found to be active against pathogenic fungi and showed higher positive inhibition than *Oudemansiella canarii, Agaricus bisporus*, and *Candida* species [98,99].

Three fungal proteins, Ganodermin (15 kDa), Agrocybin (9 kDa), and Eryngin (10 kDa), were isolated from *Ganoderma lucidum, Agrocybe cylindracea*, and *Pleurotus eryngii* mushrooms. Ganodermin actively inhibited the growth of pathogenic *Botrytis cinerea* (MIC ~15 µm), *Fusarium oxysporum* (MIC ~12 µm), and *Physalospora piricola* (MIC ~18 µm). Agrocybin exerted antifungal activity against several fungal species, but lacked inhibition against bacteria while successfully hindering HIV-1 reverse transcriptase activity. The N-terminal sequence of Eryngin demonstrated similarities with the antifungal protein derived from the *Lyophyllum shimeiji mushroom* and the similarity with thaumatin and thaumatin proteins [100–102]. A cytotoxic antifungal protease was isolated and purified from *Cordyceps militaris* with a molecular weight of 12 kDa using anion exchange chromatography on a diethylaminoethyl-sepharose column. The protein exhibited antifungal effects against *Fusarium oxysporum* and cytotoxicity effects against human breast and bladder cancer cells [90]. In another study, the plectasin peptide and 2-aminoquinoline obtained from *Pseudoplectania nigrella* and *Leucopaxillus albissimus* showed high antimicrobial activity against Gram-positive and Gram-negative bacteria, respectively [99]. In addition, the antibacterial activity of the *Lentinus edodes* mushroom against pathogenic *Staphylococcus aureus* has been reported. The oxalic acid compound present in the broth extract of the fungus was responsible for its antimicrobial effect against several other microorganisms [103].

*In vivo* animal models and *in vitro* preclinical studies showed the antibacterial and antiviral activities of *Ganoderma* mushrooms. Clinical studies indicated that *Ganoderma lucidum* polysaccharides could significantly decrease serum levels of Hepatitis B virus DNA and hepatitis B e-antigen (HbeAg) in animals, but human models still need to be supported [104]. The antimicrobial activities of 20 species of basidiomycete extracts were examined, in which the chloroform extract of *Hygrophorus agathosmus* (MIC ~7-250 μg/mL) and the dichloromethane extract of *Suillus collinitus* (MIC ~30-250 μg/mL) were found to be more potent against yeast and bacteria [105]. Furthermore, the high antimicrobial potential of *Inonotus hispidus extracts* against disease-causing *Bacillus cereus, Pseudomonas aeruginosa*, and *Candida albicans* microorganisms was evaluated and reported [59]. Similarly, ten wild mushrooms were subjected to antioxidant and antimicrobial investigation, of which the ethanolic extracts of *Pleurotus ostreatus* and *Meripilus giganteus* showed the maximum inhibitory activity (MIC ~60 μg/mL) against both bacteria and yeast [106]. A study on the methanolic extract of *Phellinus* showed that the maximum and minimum antibacterial activities against *Pseudomonas aeruginosa* varied with the extract concentrations (MIC ~50-250 μg/mL). The extract showed maximum antifungal activity against *Aspergillus flavus* and minimum against *Penicillium sp*. (MIC ~50-250 μg/mL) [107].

In a survey among 13 types of wild mushroom species, *Russula delica* and *Fistulina hepatica extracts* were found to inhibit the growth of three Gram-negative bacteria as well as six Gram-positive bacteria (MIC ~0.25–20 mg/mL). Other selected extracts of *Ramaria botrytis, Lepista nuda*, and *Leucopaxillus giganteus* also exhibited significant bactericidal effects against pathogenic bacteria (MIC ~10-20 mg/mL) [108]. The antimicrobial assay of wild mushrooms on multiresistant microorganisms showed higher synergistic effects of the *Mycena rosea* and *Fistulina hepatica* extract mixture against methicillin-resistant *Staphylococcus aureus* than against *E. coli* (MIC ~10-20 mg/mL), while the antimicrobial efficiency of *Russula delica* against *E. coli* was higher than that of *Leucopaxillus giganteus*, showing that some mushroom extracts can extensively potentiate the action of antibiotics [109].

Mushrooms possess mechanisms of action in the gut microbiota by the activity of polysaccharides, which act as prebiotics in the digestive system. The combined properties of mushroom polysaccharides are useful against chronic human diseases [89]. Chickens affected with avian *Mycoplasma gallisepticum* were treated with *Lentinus edodes* and *Tremella fuciformis* fungi supplemented with herb polysaccharides from *Astragalus membranaceus* Radix and compared with a standard antibiotic, Apramycin. Mushroom extracts stimulated the increase in potentially beneficial bacteria, thus reducing the number of potentially harmful bacteria. *Lentinus edodes extract* was associated with beneficial bacteria that lead to greater body weight gain, total aerobe, and anaerobe counts in chickens [110]. Similarly, *Astragalus membranaceus*, together with the aforementioned mushrooms, was tested for growth performance and organ weights and the gastrointestinal tract of broiler chickens. Birds that were fed the extracts showed better growth performance, and specifically, *Lentinus edodes* appeared to be a potential growth promoter [111]. *Agaricus bisporus* was compared with flavophospholipol (antibiotic growth powder) through *in vivo* studies in chickens. The results showed that the conversion ratio of the food was low in chickens fed flavophospholipol, but the mushroom-supplied diet showed a positive influence on broiler immune responses without harmful effects [112]. The general antibiotic nature of the mushrooms showed effective inhibition against bacteria and fungi to a greater extent. Thus, detailed studies indicate that this unknown source of mushroom-derived pharmaceutics can be used effectively in the manufacture of antibiotic and antimicrobial drugs.

### 4.5 Immunomodulatory and hepatoprotective effects

Most of the low molecular weight secondary metabolites derived from mushrooms possess immunomodulatory characteristics. Polysaccharides obtained from mushrooms are well-known immune potentiators to boost the immune system, specifically the one isolated from the mushroom sclerotia. One of the striking immunomodulatory actions of mushroom polysaccharides is the induction of apoptosis in tumor cells. The lymphocyte transformation test confirmed the immunostimulatory activity of the mycelia and the fruit bodies of mushrooms [113]. A study of *Agaricus blazei* reported that the mushroom possessed immunomodulatory effects such as macrophages and neutrophil activation, resulting in tumor regression [93].

Immunostimulatory activity of mushrooms is also achieved by conjugation of polysaccharide-protein complexes through an intricate mechanism. The water-soluble polysaccharide-protein mixture obtained from the *Ganoderma lucidum* spore showed its characteristic immunomodulatory activity by the proliferative response of splenocytes, thus inhibiting the growth of Lewis lung cancer cells [94]. Many clinical studies have indicated that ethanol or water extracts of medicinal mushrooms could be used against deleterious immunomodulatory effects [114]. The aqueous extract of *Inonotus obliquus* was tested on immunosuppressed mice to examine changes in immunomodulatory effects. Oral administration of the extract increased serum levels of IL-6. Control mice were chemically treated and examined with an elevated level of TNF-*α*. As a result, extract-treated mice showed higher efficiency of beneficial immunomodulatory effects than control [115]. Ergosterol peroxide (EPO), a bioactive compound present in mushroom lipopolysaccharide, induced immunomodulatory activities in human monocytic cells. The EPO complex blocked the expression of MyD88 and VCAM-1 and inhibited cytokine production [116]. In summary, many studies clearly indicated the immunomodulatory and immunostimulatory effects of mushrooms, which are highly beneficial for nutrition, growth, and disease prevention/treatment in humans and animals.

Mushrooms have traditionally been used in the treatment of liver-related diseases and ailments. Among the wide range of mushrooms under study, *Ganoderma lucidum* is undoubtedly the species most commonly considered for the treatment of liver diseases due to its beneficial hepatoprotective effects. The peptides isolated from *Ganoderma lucidum* were evaluated against d-galactosamine (d-GalN)-induced liver injury in mice liver. The biochemical results showed a significant decrease in the activity of superoxide dismutase and glutathione in the liver, which indicates the hepatoprotective action of *Ganoderma lucidum* at medium dose against hepatocellular injury [117]. In addition, *Antrodia cinnamomea*, a native Taiwan mushroom, has been reported for the treatment of hepatitis, hepatocarcinoma, and alcohol-induced liver diseases [118]. The hepatoprotection offered by *Morchella esculenta* mushroom on ethanol-induced chronic hepatotoxicity was assessed by identifying liver function marker enzymes and its antioxidant status. The mushroom extract reduced the increase in serum levels and reinstated the decreased levels of antioxidants in the ethanol-affected liver, which highlighted the hepatoprotective activity of the mushroom [119]. A similar study was performed using a wild mushroom *Macrocybe gigantea* for carbon tetrachloride (CCl_4_) induced liver damage in mice. Reduced levels of superoxide dismutase (SOD), catalase (CAT), and reduced glutathione (GSH) were restored by mushroom extract [120]. The residue polysaccharides of *Cordyceps militaris* were investigated for their pharmacological effects on lipid metabolism and oxidative stress. The residue polysaccharide, which contained glucose, arabinose, and mannose, reduced lipid levels with an improvement in glutamate pyruvate transaminase levels. Histopathological annotations established the hepatoprotective potential of *Cordyceps militaris* [121]. Thus, the bioactives of the selected mushrooms, both in edible and wild variants, exhibit efficient hepatoprotective action against liver injury and damages.

### 4.6 Anti-neurodegenerative – neuroprotective activities

Oxidative stress plays a crucial character in major neurodegenerative diseases (such as multi-cognitive impairment, dementia, etc.) by creating stress in cells and genes. Low levels of stress do not produce chronic responses, and hence the cellular mechanisms fundamental to neuroinflammatory pathogenesis are influential to Alzheimer’s disease. Mushrooms are a rich source of antioxidants and effectively mitigate the oxidative stress phenomenon in cells and neurons. The importance of nutritional mushrooms *Coriolus* and *Hericium* in reducing neuronal oxidative stress and ensuring neuroprotection in the inflammasome pathway has been reported [122].

Bioactive macromolecules in mushrooms such as hericenones, erinacines, scabronines, and dictyophorines promote the synthesis of nerve growth factors and protect the brain from neurodegeneration by inhibiting the creation of beta-amyloids and hyperphosphorylated tau and acetylcholinesterase. Mushroom extracts and their isolated compounds have many important health benefits, including immune-modulating effects on brain cells [123]. Numerous studies on culinary and medicinal mushrooms have established their neuroprotective effects with the ability to prevent neuronal death and control neurodegenerative diseases and neurotrauma [124]. Both edible and wild mushrooms play an imperative role in the prevention of many age-related neurological disorders. Some of these mushrooms, such as *Hericium, Ganoderma, Sarcodon, Antrodia, Pleurotus, Lignosus*, and *Grifola*, are used in the treatment of nervous system diseases (such as neurotoxicity) and ensure the protection of the nervous system [125,126]. In a study conducted on *Basidiomycetes, Ascomycetes*, and *Hericium* mushrooms, it was reported that these mushrooms exhibit a wide range of health benefits, such as immunological, anticancer, neuroprotective, nephroprotective, and characteristics due to the presence of various biologically active ingredients with many pharmaceutical properties [127]. Future studies of mushrooms and their bioactives are crucial in order to supplement pharmaceutical ingredients for the treatment of targeted neurodegenerative disorders.

### 4.7 Anti-diabetic and anti-hyperlipidemic activities

**Figure 3.**
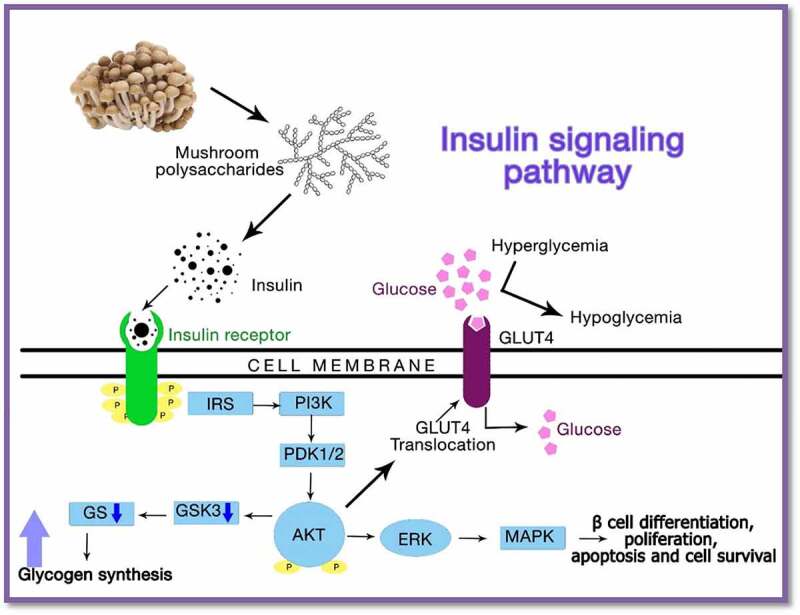
Action of mushroom polysaccharide acting on the insulin receptor thereby altering the insulin signaling pathway

In general, the homopolysaccharides present in mushrooms affect insulin metabolism by altering insulin secretion in the hormone signaling pathway (Figure 3). These health-promoting polysaccharides have been reported for their anti-obese, antidiabetic, anticarcinogenic, antimicrobial, and antiviral effects in fat cells, rodents, and humans [89]. *Ganoderma lucidum extracts*, which contained bioactive polysaccharides, proteoglycans, proteins, and triterpenoids, were administered as a substitute adjuvant for diabetes. The results showed better hypoglycemic activity by increasing plasma insulin levels and lowering plasma sugar levels in mice. *In vitro* inhibition of tyrosine phosphatase, 1B enzyme, and triterpenoids on aldose reductase and α-glucosidase by *Ganoderma lucidum proteoglycan* subdued postprandial hyperglycemia. Therefore, the hypoglycemic effects of the phytoconstituents derived from *Ganoderma lucidum* are beneficial in diabetic treatment [128,129].

Furthermore, a study on the antidiabetic effect of *Agaricus bisporus* was carried out in male Sprague-Dawley rats, which were induced with streptozotocin for type 2 diabetes. Rats fed with the mushroom supplemented food showed a significant reduction in the triglyceride (TG) concentration with high levels of antioxidants, dietary fibers, and vitamins like C, D, and B_12_ which regulated the insulin system and inhibited the diabetic disorder effects [130]. Similarly, the antihyperglycemic activity of polysaccharides was studied in *Pleurotus florida* and *Lignosus rhinocerotis* in Sprague-Dawley rats induced by hyperglycemia. Treatment with *Pleurotus florida* polysaccharides significantly reduced glucose, serum cholesterol, triglycerides, and ketones in animals. The study concluded that polysaccharides could improve hyperglycemia and hypercholesterolemia, including other complications in the therapy of type 2 diabetes mellitus [131,132]. A comparative study on six edible and medicinal mushrooms was reported for their antidiabetic potential in terms of the inhibitory action of the α-amylase and α-glucosidase. Inonotus obliquus promoted maximum inhibition, while *Morchella conica* and *Cordyceps militaris* did not show any inhibition. This investigation provided information on the different mushroom species showing their effective activity for type 2 diabetes treatment [133]. Thus, the broad spectrum of antidiabetic action of the bioactive mushrooms, predominantly through the enhancement of insulin secretion, marks their suitability for the production of diabetic drugs.

Edible and medicinal mushrooms belonging to different species such as *Agaricus, Ganoderma, Grifola, Hericium, Lentinus, Pholiota, Pleurotus*, etc., were found to lower blood cholesterol and triglyceride levels. Lovastatin, a statin produced by *Omphalotus olearius* and *Pleurotus ostreatus*, strongly inhibited hydroxymethylglutaryl coenzyme A reductase, an important enzyme in the cholesterol biosynthesis pathway. Similarly, chitosan isolated from *Ganoderma lucidum* and its fibers proved its potential to decrease blood lipid levels [134]. Different dosages of various extracts of the edible mushrooms *Pleurotus ferulae, Pleurotus citrinopileatus*, and *Lentinus lepideus* were studied in hyperlipidemic rats. The results of the dose-dependence studies for all mushrooms showed a reduction in the levels of triglycerides and total cholesterol. Furthermore, the levels of high-density lipoproteins were moderate to high, revealing the antihyperlipidemic potential of mushrooms [135–137]. The oyster mushroom, *Pleurotus ostreatus*, which was found to decrease lipid levels in animals, was subjected to human trials in HIV patients. Small changes in the levels of high-density lipoproteins and triglycerides were observed, although a significant result of clinical magnitude was not observed [138]. The hypolipidemic effect of *Pholiota nameko* polysaccharide (PNPS-1) was studied in hyperlipidemic Wistar rats. PNPS-1 was found to be effective in lowering the body weight of treated rats and also improved the pathologic changes in the coronary arteries of hyperlipidemic rats that showed its therapeutic potential against hyperlipidemia [96]. Most medicinal mushrooms have effective antihyperlipidemic activity in both animals and humans, indicating their potential to be used as a nutraceutical to enhance the lipid profile.

### 4.8 Anti-hypertensive and cardioprotective effect

Cardiovascular issues are one of the leading health risks among the most common reasons for mortality. Fatty acids, cholesterol, lipoproteins, and triacylglycerols are regulated by the bioactives of mushrooms, which efficiently reduce the threat of cardiovascular diseases [22]. Mushrooms demonstrate their cardioprotective actions through their bioactives on metabolic markers such as low-density lipoprotein (LDL), high-density lipoprotein (HDL), and homocysteine levels, which are associated with cardiovascular disorders [139]. In a study among nine edible mushrooms, *Pleurotus cystidiosus* recorded the highest antihypertensive activity due to a protein of molecular weight ~8300 Da present in it [140]. In another study, proteins extracted from *Pleurotus cystidiosus* (E1Pc and E5Pc) and *Agaricus bisporus* (E1Ab and E3Ab) had high levels of antihypertensive activity with a variety of high to low molecular weight proteins in the range of 3 to 10 kDa [141]. The cardioprotective effects of *Ganoderma lucidum* extract were examined on perfused rat hearts with global ischemia for 45 min and reperfusion for 30 min. The results showed the cardioprotective effect of the mushroom extract due to its antioxidant properties, which reduced the necrotic death of cardiomyocytes and reperfusion contracture [142]. In another study, the cardioprotective role of *Ganoderma lucidum* was evaluated against Adriamycin-induced toxicity in Wistar rats. The extract exhibited momentous antioxidant properties to protect the rat heart from the toxicity of Adriamycin [143].

Different fractions of hot water extract of *Pleurotus nebrodensis* were tested in single-dose and continuous-dose modes in hypertensive rats. Studies concluded that single-dose treatment decreased systolic blood pressure due to the cardioprotective effects of the polysaccharides and proteins present in the mushroom extract [144]. The ethanolic extract of *Astraeus hygrometricus* was tested on cardiomyocytes *in vitro*, which showed a correlation with hypertrophy and potent antiapoptotic effects. This hypertrophy was directly related to an increase in the expression of proto-oncogenes, hypertrophy marker genes, growth factors, and cytokines that mimic human cardiomyopathy [145]. In general, the antihypertensive and cardioprotective effects of various edible and wild mushrooms highlight their potential to be used in the treatment of cardiovascular disease and other heart-related diseases.

### 4.9 Nephro, renoprotective and diuretic effect

The chemoprotective effects of the hot water extract of *Pleurotus tuberregium* were evaluated in the liver model and kidney toxicity induced by carbon tetrachloride (CCl_4_) and paracetamol in untreated rats. Serum creatinine and urea concentration were observed to decrease with an increase in antioxidant enzymes, which exhibited the renoprotective effects of *Pleurotus tuberregium* due to oxidative impairment of the kidney, initiated by drugs and toxicants [146]. Different assays of the *Pleurotus eous* extract were tested for *in vitro* antioxidant potential and nephroprotective activity against renal failure induced by cisplatin administration in Swiss albino mice. The extracts were able to regenerate renal cells in mice, establishing the nephroprotective nature of *Pleurotus eous* [147]. In a study of *Ganoderma lucidum*, the medicinal values of the mushroom were reported for the prevention of a wide range of diseases, including diabetic nephropathy. Renoprotective effects of the new meroterpenoids, chizhines A–F (1–6), were identified to inhibit monocyte chemotactic protein 1 (MCP-1) and fibronectin synthesis in high glucose-induced rat mesangial cells [148].

In another study, approximately 12 phenolic meroterpenoids, cochlearols, and ganocochlearins were isolated from *Ganoderma cochlear* to examine their renoprotective potential in renal interstitial fibroblast cells (NRK-49 F) of rats. Phenol meroterpenoids displayed strong inhibitory actions against fibronectin in TGF-β1-induced NRK-49 F cells [149]. The methanolic extract of the fruiting bodies of *Pleurotus cornucopiae* showed renoprotective effects against cisplatin-induced damage to kidney cells. Ten of the 12 isolated compounds repudiated the damage induced by cisplatin-induced LLC-PK1 cells, while the other few compounds improved cisplatin-induced nephrotoxicity to 80% [150]. In another work, Poria mushrooms were investigated for their antioxidant and renoprotective effects against oxidative stress exerted by CaO_x_ monohydrate or H_2_O_2_ in renal cells. Despite the oxidative stress caused in the G_1_ cell cycle, the extract was powerful enough to prevent cellular effects arbitrated by oxidative stress [151]. *Lentinula edodes* demonstrated high anti-inflammatory effects to decrease serum levels of TNF-α, IL-6, and IL-1β and to improve the antioxidant eminence of renal enzyme actions for the treatment of LPS-induced kidney injury in mice. The renoprotective behavior of the *Lentinula edodes* polysaccharides in the renal cortex and renal medulla highlighted its efficacy for the treatment of kidney injuries [152]. Thus, aqueous and organic extracts of mushrooms ensure their nephroprotective and renoprotective effects, allowing them to be used in the effective treatment of kidney ailments and injuries.

## 5. Challenges and future prospects

Edible mushrooms are natural and healthy food sources that provide distinctive biological and functional benefits. The unique medicinal characteristics of the various mushrooms provide them with both nutritional and economic values [153]. Of the various pharmacological characteristics of different mushrooms examined in this review, the top three variants for each of the medicinal characteristics are tabulated in Table 2. From the table, it can be seen that *Ganoderma lucidum* and *Hericium erinaceus* cover almost all the pharmacological attributes, making them highly preferred for cultivation and medicinal applications.

**Table 2.** Top 3 mushrooms for various pharmacological effects of mushrooms

**S.No**	**Medicinal Property**	**Mushroom**	**Minimum** **concentration**	**Reference**
1	**Antioxidant activity**	*Inonotus obliquus*	5 mg/mL	[61]
		*Ganoderma lucidum*	1 mg/mL	[54]
		*Agaricus bisporus*	6 GAE/mL	[60]
2	**Anti-inflammatory activity**	*Cordyceps militaris*	300 µg/mL	[73]
		*Hericium erinaceus*	0.5 mg/L	[75]
		*Phellinus igniarius*	100 μmol/L	[231]
3	**Anticancer activity**	*Ramaria flava*	200 µg/mL	[91]
		*Phellinus linteus*	250 µg/mL	[86]
		*Pleurotus ostreatus*	500 µg/mL	[88]
4	**Antibiotic activity**	*Agrocybe aegerita*	-	[232]
		*Ramaria flava*	2 mg/mL	[91]
		*Lentinus edodes*	-	[103]
5	**Immunomodulatory potential**	*Pleurotus ostreatus*	20 µg/mL	[201]
		*Grifola frondosa*	25 µg/mL	[191]
		*Lactarius deliciosus*	30 µg/mL	[203]
6	**Neuroprotective effect**	*Hericium erinaceus*	100 µg/mL	[127]
		*Phellinus rimosus*	50 µg/mL	[207]
		*Cantharellus cibarius*	100 µg/mL	[177]
7	**Antidiabetic property**	*Ganoderma lucidum*	100 µg/mL	[54,128,129]
		*Inonotus obliquus*	50 µg/mL	[233]
		*Pleurotus florida*	400 µg/mL	[131]
8	**Cardioprotective effect**	*Ganoderma lucidum*	400 µg/mL	[142,143,234]
		*Agaricus brasiliensis*	200 µg/mL	[235]
		*Cordyceps sinensis*	10 µg/mL	[236]
9	**Hepatoprotective effect**	*Ganoderma lucidum*	180 µg/mL	[237]
		*Cordyceps militaris*	400 µg/mL	[121]
		*Morchella esculenta*	500 µg/mL	[119]
10	**Nephroprotective effect**	*Pleurotus eous*	500 µg/mL	[147]
		*Ganoderma lucidum*	500 µg/mL	[148]
		*Pleurotus ostreatus*	200 µg/mL	[238]

In addition to nutritional and health supplementation impacts, mushroom cultivation has wider roles in terms of alleviating poverty, providing livelihood and employment opportunities, mitigating environmental degradation, etc. The salient characteristics and biological functionalities of the various bioactives (especially polysaccharides, proteins, and antioxidant compounds) present in wild edible mushrooms have generated great interest in their cultivation [18]. As these variants thrive in wild environments, their commercial cultivation should involve local communities. In addition, these communities would play a crucial role in the establishment of ethnomycological information and the creation of a comprehensive knowledge database for edible wild mushrooms. Proper policies and systems must be developed to train these communities to ensure equitable economic growth and socio-economic impact through mushroom cultivation. Also, research studies on the effect of cultivation conditions, substrate composition, and harvest time on the phytochemical composition and nutritional value of mushrooms must be carried out in detail to achieve effective cultivation of wild edible mushrooms.

Furthermore, more research efforts are required to fully understand the mechanism and metabolic pathways of mushroom bioactives for their pharmacological activities. Both wild and cultivated mushrooms consist of metals and trace elements like cadmium (Cd), selenium (Se), chromium (Cr), lead (Pb), arsenic (As), mercury (Hg), zinc (Zn), and nickel (Ni) among which some elements are toxic to humans if consumed daily [154–156]. Mleczek et al., in a very recent study, reported different elements present in four wild-type mushrooms (*Boletus edulis, Imleria badia, Leccinum scabrum*, and *Macrolepiota procera*) and concluded that over intake of mushrooms may result in health risks to humans [157]. Also, the preservation procedures like drying and freezing have a few negative effects on the composition and characteristics of mushrooms, such as degradation of proteins, polysaccharides depolymerization, loss of amino acids, and vitamins degradation. Research studies on the processing and preservation methods for mushroom storage must be explored to ensure longer shelf life and loss of resources. Changes in the phytochemical composition and alterations in the mechanisms of bioactives during the processing and storage of mushrooms must be investigated. In addition, genome sequencing and the application of advanced technologies such as metabolomics, proteomics, transcriptomics, etc., can provide valuable insights and novel ideas for the research, categorization, and cultivation of medicinal mushrooms [19]. More research works on gene alterations, and novel processing technologies are required for the production and processing of mushrooms which would be more safe, nutritive, and therapeutic for human health.

Despite the promising medicinal and therapeutic potential of the mushrooms, the pharmacological applications are not sufficiently commercialized. More efforts are needed on the exploration of the therapeutic potential of medicinal mushrooms and drug development. Essential tasks include successful human-based clinical studies using high-quality mushroom-derived products for disease treatment and establishing economical ways to produce these products under controlled conditions [17]. Recently, in addition to conventional medicines, supplementary medicines are becoming popular among patients to manage their distress during and after treatment. Supplemental non-pharmacological treatments utilizing medicinal mushrooms have limited data on their cost and benefits. Thus, to expand the knowledge in this field and facilitate the comprehensive assessment of mushrooms’ therapeutic benefits, more research is needed in preclinical and clinical studies based on relevant data.

### 6. Conclusion

Mushrooms are widely explored for their medicinal traits and supplemented as an important dietary product along with standard drugs and therapeutics. Polysaccharides, oligosaccharides, dietary fibers, peptides, amino acids, fatty acids, micronutrients, and phenolic bioactives present in mushrooms impart a wide spectrum of medical and pharmacological properties, which have been established through several *in vitro* and *in vivo* studies on animal and human models. These components follow complex pathways to exhibit functional and biological activities, individually or synergistically. These higher classes of fungi show various medicinal properties, namely antioxidant potential, anti-inflammatory activity, and anti-aging, which are attributed to their fiber and polysaccharide content. Other dietary fibers and saccharides act against hypertensive, hyperlipidaemic, diabetic, and adverse immunomodulatory conditions. The terpenoids and phenolic compounds in the mushroom are responsible for the protective actions on the heart, liver, neurons, kidneys, and liver. The cytotoxic nature of mushrooms against cancer and tumor cells proves the bioactive constituents of mushrooms as an emerging natural pharmaceutical. Future prospects on mushroom research can involve the development of new cultivation technologies and post-harvest processing methods to overcome the issues and challenges associated with mushroom plantations. Also, more research studies, including complete human clinical trials, must be performed to understand the mechanism and metabolic pathways of mushroom bioactives interactions for their pharmacological activities and generate relevant data. More detailed studies on the unexplored wild edible variants and their cultivation conditions are required to tap the complete potential of mushrooms for the benefit of human health and life.
